# A miniature fluorescence microscope for multi-plane imaging

**DOI:** 10.1038/s41598-022-21022-9

**Published:** 2022-10-06

**Authors:** Giovanni Barbera, Rachel Jun, Yan Zhang, Bo Liang, Yun Li, Da-Ting Lin

**Affiliations:** 1 Intramural Research Program, National Institute on Drug Abuse, National Institutes of Health, Baltimore, MD 21224 USA; 2 School of Electrical Engineering and Computer Science, University of North Dakota, Grand Forks, ND 58202 USA; 3 Department of Zoology and Physiology, University of Wyoming, Laramie, WY 82071 USA; 4 The Solomon H. Snyder Department of Neuroscience, Johns Hopkins University School of Medicine, Baltimore, MD 21205 USA

**Keywords:** Neural circuits, Microscopy

## Abstract

Miniature fluorescence microscopes are becoming an increasingly established tool to investigate neural circuits in freely moving animals. In this work we present a lightweight one-photon microscope capable of imaging at different focal depths. The focal plane can be changed dynamically by modulating the pulse width of the control signal to a variable focus liquid lens, which is synchronized to the image sensor to enable changing focal plane between frames. The system was tested by imaging GCaMP7f expressing neurons in the mouse medial prefrontal cortex (mPFC) in vivo during open field test. Results showed that with the proposed design it is possible to image neurons across an axial scan of ~ 60 μm, resulting in a ~ 40% increase of total neurons imaged compared to single plane imaging.

## Introduction

Over the past decade, the development of miniature fluorescence microscopes (commonly referred to as miniscopes), paired with genetically encoded calcium indicators and voltage sensitive dyes has grown to be one of the most powerful tools available in neuroscience^1–5^, enabling researchers to investigate neural circuits in vivo and correlate the activity of large neuronal populations with complex behaviors which could not be replicated in head fixed animals, in timescales spanning from days to months^6,7^.

These versatile tools offer a unique insight into key neural processes involved in complex behaviors or animal models of disease, fueling the development of new generation of miniscopes to expand the horizon of neural circuits investigation. Active areas of research include simultaneous imaging and optogenetic stimulation of targeted neurons^8–10^, to untangle causality of neuronal population activity in specific behaviors; wireless miniscopes^11,12^, to reduce the influence of a tether on the animal’s behavior, and to enable neural activity recording in more complex behaviors (such as multiple subject social interaction); other versions of miniscopes include large scale imaging^13–15^, and 2-photon for imaging at multiple depths^16–19^.

A key element for expanding the miniscope’s potential is the ability to change focal plane rapidly and reliably during experiments. This would allow not only a more stable focusing system, improving the quality of individual neurons’ tracking over time, but also imaging on different focal planes, enabling multi-color imaging. There are several methods which can be leveraged for achieving this goal in multi-photon imaging (piezoelectric actuators, voice coil-motors actuators, tunable acoustic gradient index lenses^20^), however these techniques are not easily scalable to miniaturized, single photon microscope for studying neural circuits in freely behaving rodents. Electrically Tunable Lenses (ETLs, or liquid lenses) can be more effectively integrated in such applications^20–23^. Bagramyan et al. developed a tunable liquid crystal lens^24,25^ which was integrated in a one-photon miniscope for sub-cellular resolution imaging. A benchtop two-photon implementation of a remote focusing system based on liquid lens was described in^26^, but limited to head-restrained behaviors. An expansion to the UCLA miniscope also includes an ETL^15^, however the total weight is 13.9 g, which makes it too heavy to be used in freely moving mice.

In this study we present an open-source miniature miniscope capable of remote and dynamic focal plane change leveraging a commercially available variable focus liquid lens, which can be easily integrated in custom imaging systems.

## Methods

All experimental procedures and animal care were reviewed and approved by the Institutional Animal Care and Use Committee (IACUC), the Intramural Research Program, National Institute on Drug Abuse, National Institutes of Health. All animal studies were carried out in accordance with guidelines of the approved IACUC protocol and in accordance with ARRIVE guidelines.

### Viral injection

The viral injection procedure was similar to the one described previously^27^, and briefly outlined here. Six C57BL/6 J male mice (age of 3–4 months, body weight ~ 25 g) were injected with AAV-pgk-Cre (Addgene, #24593-AAVrg) into Nucleus Accumbens and pGP-AAV-syn-FLEX-jGCaMP7f-WPRE (Addgene, #104492-AAV1) into prelimbic cortex (PrL). Briefly, mice were anaesthetized with 2% isoflurane in oxygen at a flow rate of 0.4 L/min and mounted on a stereotactic frame (Model 962, David Kopf Instruments), while body temperature was maintained at 37 °C using a temperature control system (TCAT-2DF, Physitemp). Sterile ocular lubricant ointment (Dechra Veterinary Products) was applied to mouse corneas to prevent drying. A hole was drilled through the right side of the skull above the injection site (A/P: + 1.9 mm; M/L: − 0.3 mm) using a 0.5-mm diameter round burr on a high-speed rotary micro drill (19007-05, Fine Science Tools). A total of 500 nl of virus (a titer of 6.75e12 GC/mL) was injected using the stereotactic coordinates (A/P: + 1.9 mm, M/L: − 0.3 mm, D/V: − 1.7 mm, 0° angle) at a rate of 25 nl/min with a micro pump and Micro4 controller (World Precision Instruments). After injection, the injection needle was kept in the parenchyma for 5 min before being slowly withdrawn. The hole on the skull was then sealed with bone wax, and the skin was sutured. After surgery, Neosporin ointment was applied to the closed skin incision line. Mice were subcutaneously injected with buprenorphine (0.05 mg/kg) and returned to their home cage to recover from anesthesia in a 37 °C isothermal chamber (Lyon Technologies, Inc). Mice were maintained on ibuprofen (30 mg/mL in water) ad libitum for at least 3 days post-surgery.

### Gradient index (GRIN) lens implantation

The GRIN lens implantation procedure was similar to the one described previously^27^, and briefly outlined here. One week after viral injection, a 1-mm diameter gradient index (GRIN) lens (GRINTECH GmBH) was implanted in the mouse brain in the mPFC. Briefly, mice were anesthetized with ketamine/xylazine (ketamine:100 mg/kg, xylazine:15 mg/kg), and a 1 mm-diameter craniotomy was generated in the right hemisphere above the coordinates (A/P: + 1.9 mm, M/L: − 0.7 mm). Freshly prepared artificial cerebrospinal fluid (aCSF) was continuous applied to the exposed tissue throughout the surgery to prevent brain tissue dehydration. The brain tissue above the PrL, along a direction of a 10° laterally shifted angle into a depth of 1.8 mm was precisely removed using vacuum suction through a 30-gauge blunted needle attached to a custom-constructed three-axis motorized stereotactic device, modified from a commercial stereotactic frame (Model 962, David Kopf Instruments). After brain tissue above mPFC had been removed and the surgical site was clear of blood, a GRIN lens was slowly lowered into the mPFC and secured to the skull using dental cement (DuraLay). About one month after the GRIN lens implantation, a custom baseplate was mounted onto the mouse head with dental cement. The miniscope was then secured to the baseplate using 3 screws.

### Miniscope design

**Figure 1:**
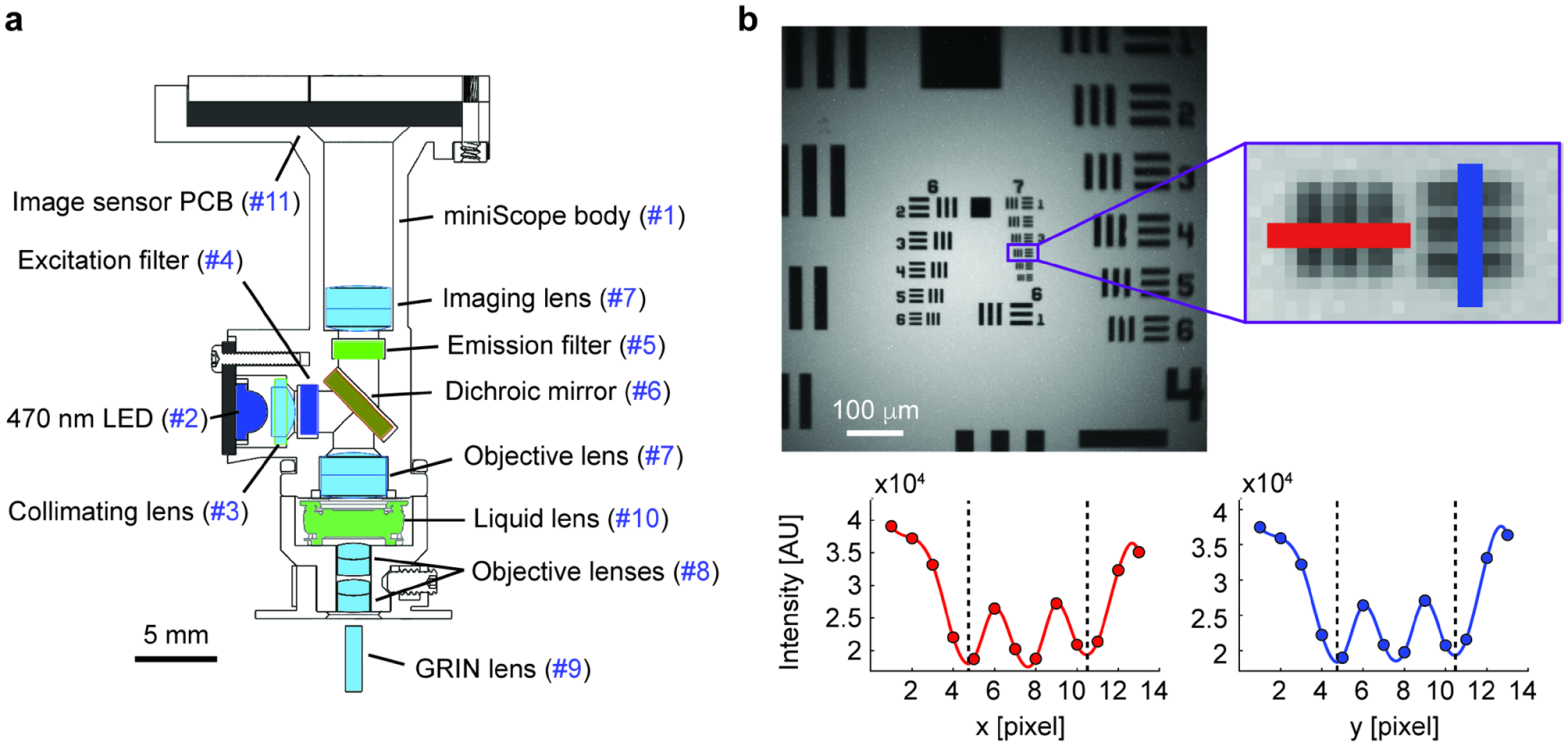
Miniscope characterization. (**a**) Schematic of the miniscope design. Numbers in blue correspond to line items in Table 1. (**b**) Top: image from reflected light on a 3″ × 3″ 1951 USAF target. Inset: zoomed-in detail of group 7, element 4 (181 lp/mm). Bottom: intensity values for the pixels highlighted in the inset row (left) and column (right), spanning two line pairs over 11.05 μm, and resulting in a 3× magnification factor with 5.52 μm resolution.

The miniscope consists of the main body, designed in Solidworks and 3D-printed in black resin (Protolabs, Maple Plain, MN), light source (high power 470 nm LED XPEBBL-L1-0000-00302, Cree LED), optics (see Table 1) and a CMOS sensor (MT9V022, Aptina/Onsemi) mounted on a custom PCB which connects to the data acquisition system as described in^28^. A schematic of the miniscope mechanical design is shown in Fig. 1a. We used a variable focus liquid lens (A-16F0-P12, Corning Varioptic), which allows for a fast change of focal length, controlled and synchronized to the miniscope image sensor through the custom FPGA control board.

**Table 1:** Bill of materials.

Item #	Manufacturer	Manufacturer P/N	Description	Qty
#1	Protolabs	Custom miniscope body	Black resin 25 μm tolerance	1
#2	Mouser	941-XPEBBLL10000302	Cree high power blue LED	1
#3	Edmund Optics	83605	LightPath 355160 | 4 mm Dia., 0.55 NA, BBAR (350–700 nm), Molded Aspheric Lens	1
#4	Chroma	ET470/40x	Excitation filter 450–490 nm, 3 × 3 × 1 mm	1
#5	Chroma	ET525/50 m	Emission filter 3 × 3 × 1 mm	1
#6	Chroma	T495lpxr	Dichroic mirror 5 × 5 × 1 mm	1
#7	Edmund Optics	63704	4 mm Dia. × 12 mm FL, VIS 0° Coated, Achromatic Lens	2
#8	Edmund Optics	84126	2 mm Dia. × 6 mm FL, VIS 0° Coated, Achromatic Lens	2
#9	GRINTech	NEM-100-25-10-860-S-0.5p	1 mm Dia GRIN lens	1
#10	Edmund Optics	A-16F0-P12	A-16F0 with FPC-A-12 variable focus Liquid Lens	1
#11	Sierra Circuits	Custom image sensor PCB	PCB hosting miniscope image sensor/LED control	1

### Benchmarking

We tested the optical performance of the miniscope by imaging a 1951 USAF target (Fig. 1b), measuring a vertical and horizontal resolution of 5.52 μm for a ~ 3× magnification factor.

**Figure 2:**
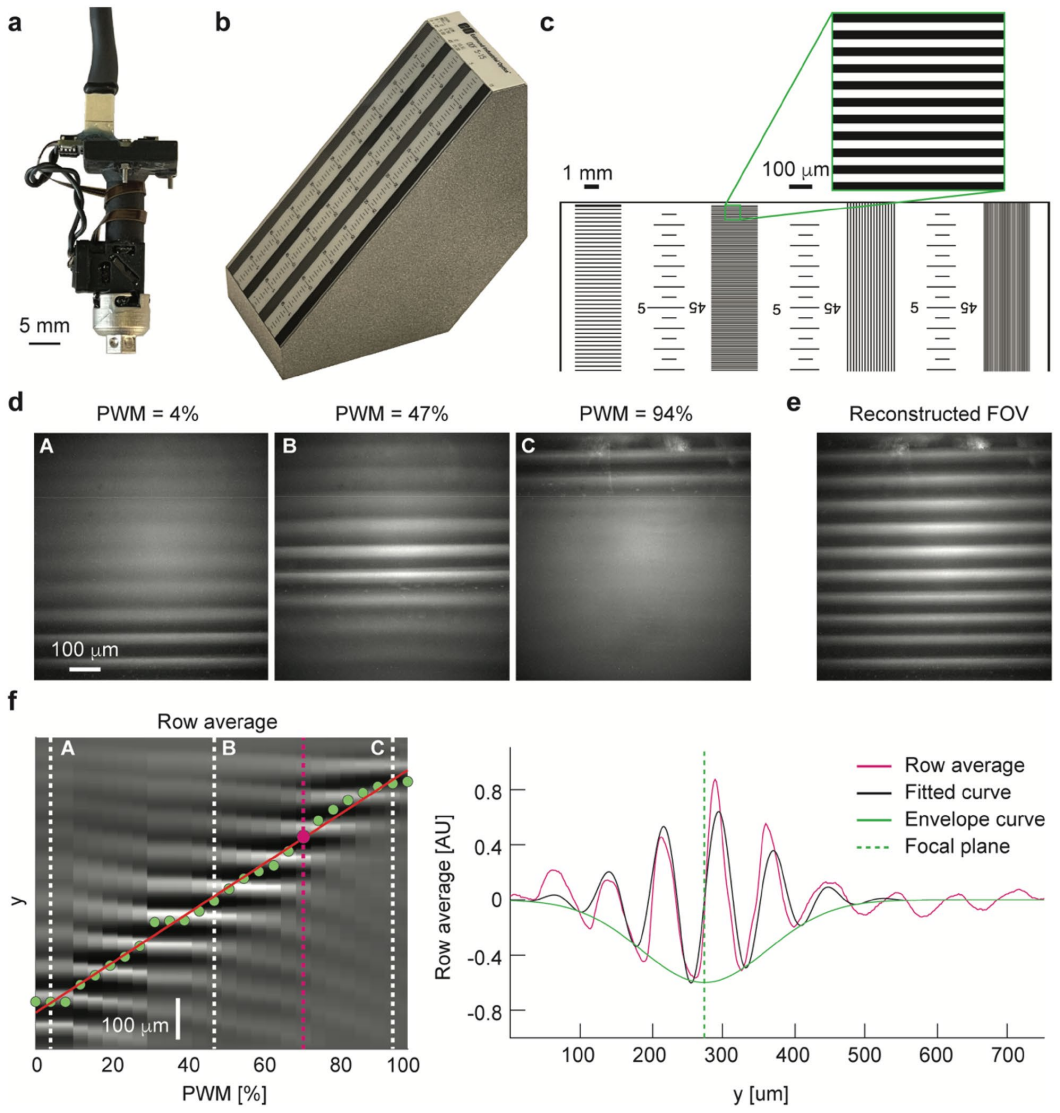
Evaluation of focal plane change. (**a**) The assembled miniscope was placed vertically on the depth of field target to evaluate its axial scanning performance. (**b**) A photo of the a 45° depth of field target. (**c**) Detail of the depth of filed target, highlighting a detail of the imaged field of view from the 15 lp/mm section (inset) (**d**) Images captured at 3 different focal plane depths on a 45° depth-of-field target with 15 lp/mm. (**e**) Reconstructed field of view (FOV), merging the 40 rows around the estimated focal distance for each depth. (**f**) Left: row average of images captured at different focal plane depths, spanning from 0 to 100% PWM cycle of the liquid lens control signal. Green dots show the estimated focal plane depth for each frame, and the red line is their linear fit (slope of 5.98 μm per percent change in PWM duty cycle). Dashed white lines represent the 3 frames in (**d**). Right: pixel row-average for the image captured at 66% PWM cycle control signal and shown in magenta dashed line in left panel. The center of the fitted exponential envelope curve (green dashed line) is used to estimate the focal plane depth.

The axial scanning performance was tested by positioning the miniscope vertically (Fig. 2a) over a 45° depth of field target (Edmund Optics DOF 5–15, Fig. 2b), and recording the horizontal lines from the 15 lp/mm section (Fig. 2c) at different plane depths, ranging from 0 to 100% PWM duty cycle of the liquid lens control signal in 26 steps (Fig. 2d,e). For each plane depth, we calculated the average of a 30-frame sequence. The change in control signal produced a change in focal plane depth which was estimated through the curve fitting parameters of the frames row-average: by averaging along y for each duty cycle step, the resulting curves were approximated as a Fourier series truncated at the first harmonic (tuned to the frequency of the line pairs, ω = 66.67), multiplied by an exponential envelope curve, whose center parameter b indicated the estimated depth of the focal plane:

$$a\cdot {e}^{-{\left(\frac{x-b}{c}\right)}^{2}}\cdot ({A}_{0}+{A}_{1}\mathrm{cos}\left(\omega x\right)+{B}_{1}\mathrm{sin}(\omega x))$$

The focal plane change as a function of the liquid lens PWM duty cycle could be approximated linearly (R^2^ = 0.99287) with a slope of 5.98 μm per percent change in duty cycle (Fig. 2f).

**Figure 3:**
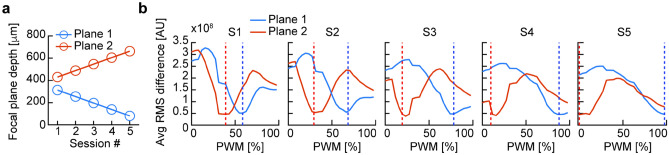
Focal plane fast switch. (**a**) Focal plane depth (estimated using the linear fit from Fig. 2c) for 5 sessions recorded alternating between two focal planes every frame, with 100 frames recorded at each depth. (**b**) Average pixel intensity RMS for the image difference between the average of 100 frames recorded at each of the alternating plane depths (red and blue for plane 1 and plane 2, respectively), and frames recorded statically across all PWM cycle control values. Dashed lines mark the PWM cycle used on each of the two planes. Local minima of the RMS difference curve denote higher similarity between the two images.

To assess whether the image could stabilize within the time between two frames, we recorded 200 frames at 10fps, alternating focal plane depth at each frame, for 5 sessions (Fig. 3a).

We then calculated the RMS difference between the 100 frame-average for each plane and the static frames captured across the 26 PWM cycle steps (Fig. 3b): for all sessions, across all focal plane differences, the local minima of the RMS curve (and the highest similarity between frames) coincided with the focal plane at which the alternating planes were recorded, indicating a close match between the static images captured at each depth and the corresponding alternating frames.

## Results

**Figure 4:**
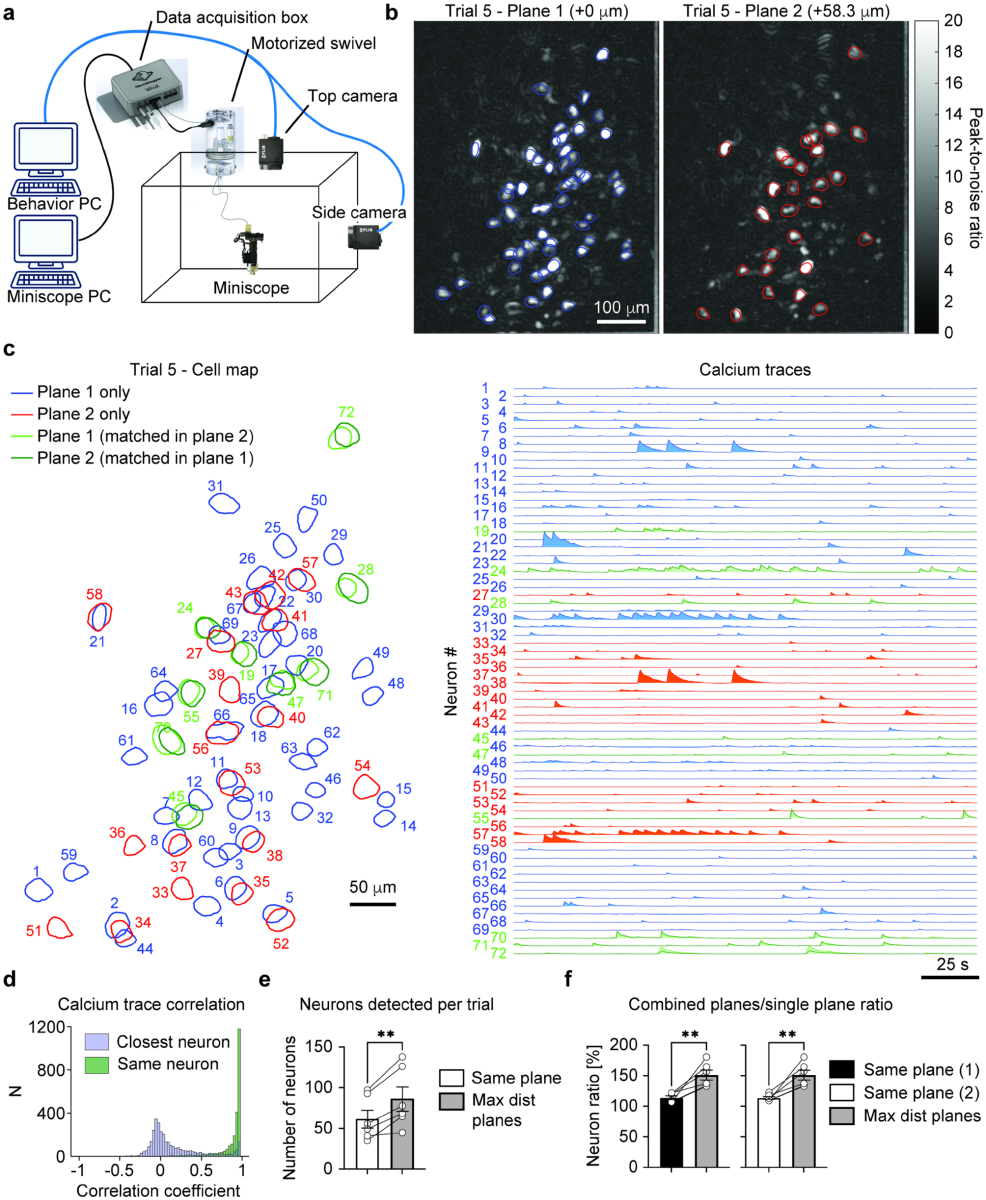
In vivo imaging of mPFC neurons expressing GCaMP7. (**a**) Scheme of the setup used for the open field recordings. (**b**) Peak-to-noise ratio for Plane 1 (left) and Plane 2 (right) in a representative trial with focal length difference of 58.3 μm. (**c**) Left: footprints of the neurons identified in the same trial shown in (**b**), highlighting neurons detected only on plane 1 (blue), only on plane 2 (red) and on both planes (light green for neurons detected in Plane 1 with a matching neuron on Plane 2, and dark green for neurons detected in Plane 2 with a matching neuron on Plane 1). Right: associated neural traces. (**d**) Histogram of the correlation coefficient for single trial calcium traces of neurons registered as same in plane 1 and plane 2. (**e**) Number of neurons detected per trial in trials with no change in focal plane and trials with maximum distance between the two focal planes (two-tailed paired t-test, *p* = 0.0053, n = 6 mice). (**f**) Ratio of total neurons detected per trial (using the information from both planes) over the average neurons detected per plane in the same trial. Data is shown separately for same focal plane at offset = 0 μm (trials 2, 6 and 14, two-tailed paired t-test, *p* = 0.0069, n = 6 mice) and same focal plane at offset = 58.3 μm (trials 4, 8 and 15, two-tailed paired t-test, *p* = 0.0055, n = 6 mice).

To demonstrate the ability of both imaging at single neuron level and detecting different neurons across different focal plane depths, we performed in vivo imaging on mice (n = 6) expressing GCaMP7f in the mPFC, as described previously^28^. The setup used to carry out the recordings is depicted in Fig. 4a. Imaging was performed during 15 open field trials (sample images from top and side view camera is shown in Supplementary Fig. 1a), recording 2000 frames per trials at 10 fps, and alternating focal plane at each frame following the schedule shown in Supplementary Fig. 1b. The maximum focal depth change was limited by light scattering in the deeper brain tissue, and it was experimentally measured to be ~ 60 μm across all imaged mice.

For each trial, the two interleaved data streams of 1000 frames per plane were separately processed by performing a Fourier-based motion correction first^29^, and then applying the CNMFe based calcium trace extraction method described previously^30^. The resulting spatial components were then registered across the two imaging planes and trials with the method described previously^31^, which estimates the likelihood of two neurons from different focal planes to be the same based on a probabilistic model built on the centroid distance and spatial footprint correlation. Representative cell maps and peak-to-noise ratios (PNR) for the two focal planes at the maximum distance is shown in Fig. 4b and Supplementary Fig. 2a. Based on the cell registration results, all neurons detected in each trial were assigned to either plane 1, plane 2, or both planes (Fig. 4c, Supplementary Movie 1). Examples of neurons detected only in one plane are shown in Supplementary Fig. 2b.

Neurons identified as common ones in both focal planes showed high correlation in their activity measured in the two focal planes (Fig. 4d), confirming the same identity in both spatial and temporal components. On the other hand, calcium traces from closest non-same neurons were largely uncorrelated (Fig. 4d), with some exceptions represented by cells whose footprint differed due to focal, but likely were the same neurons. To reduce the number of false negatives in the neuron registration across focal planes, one could use a less conservative approach by including not only spatial footprint criteria, but also a temporal component matching.

Overall, we could identify a larger number of neurons in trials with maximum distance between the two focal planes compared with trials with no focal plane change (two tailed paired t test, *p* = 0.0053, Fig. 4e), for an average increase in number of detected neurons per trial of 41.67% $\pm 24.46\%$ SD in n = 6 mice.

We then measured the ratio of total number of neurons detected on each trial (merging the information from both focal planes) and the average number of neurons detected on each focal plane (Fig. 4f), which also resulted in a larger number neurons when the two focal planes were at maximal distance compared to when the same focal length was used for both planes (two tailed paired t test, n = 6 mice, *p* = 0.0069 for same plane with offset = 0 μm (trials 2, 6 and 14) and *p* = 0.0055 for same focal plane at offset = 58.3 μm (trials 4, 8 and 15).

## Discussion

We presented the design of a miniature fluorescence microscope which integrates a variable focus liquid lens, enabling a dynamic change of focal plane between frames during in vivo calcium imaging recordings. Despite the limitation in the maximum possible focal length posed by light scattering in single photon imaging, the integration of a variable focus lens offers several advantages that can push the boundaries of in vivo neural circuit investigation. Beyond increasing the number of imaged neurons, it simplifies the focusing mechanism of miniature microscopes, which often consists of rotating parts, making it difficult to reliably image the same plane and possibly introducing image registration issues across sessions.

Compared with other variable focus imaging systems, the proposed miniscope is more lightweight^15^, and it is tethered with a flexible cable which, combined with a motorized swivel, allows for less constraint on the rodent’s head than fiber coupled systems^23^.

One aspect to consider regarding the accuracy of the neuron identification in the two focal planes, is that current neuron registration algorithms mainly target session-to-session registration^6,31^, and therefore rely on spatial information only to infer the likelihood of two neurons being the same; in the multi-plane context, there is also a temporal component which can be used, as the extracted calcium traces from the same neuron in the two planes are expected to be very similar. A hybrid registration method which accounts for both spatial and temporal neural components would improve the accuracy of neuron identification in multi-plane imaging.

We believe ETLs will be integrated increasingly more in miniature single- and multi-photon microscopes due to the advantages it offers over other tunable focus technologies^20^. A potential development of the variable focus design is the application to dual-color imaging, where focal plane change between the two layers of neurons imaged with two different colors can be corrected for on alternating frames.

## Supplementary Information

Supplementary Information 1.

Supplementary Video 1.

## Data Availability

The datasets generated during and/or analyzed during the current study are available from the corresponding author on reasonable request.

## Code availability

The code and design files are available on github at https://github.com/giovannibarbera/miniscope_v2.0.
