# Supplementary material for: A miniature fluorescence microscope for multi-plane imaging
Source: Sci Rep. 2022 Oct 6;12:16686. doi: 10.1038/s41598-022-21022-9 (PMC9537509; doi:10.1038/s41598-022-21022-9)
Supplement: Supplementary file 1 — Supplementary Information 1. [file 41598_2022_21022_MOESM1_ESM.docx]

**A Miniature Fluorescence Microscope for Multi-Plane Imaging**

**Supplementary information**

Giovanni Barbera, Rachel Jun, Yan Zhang, Bo Liang, Yun Li, Da-Ting Lin

**
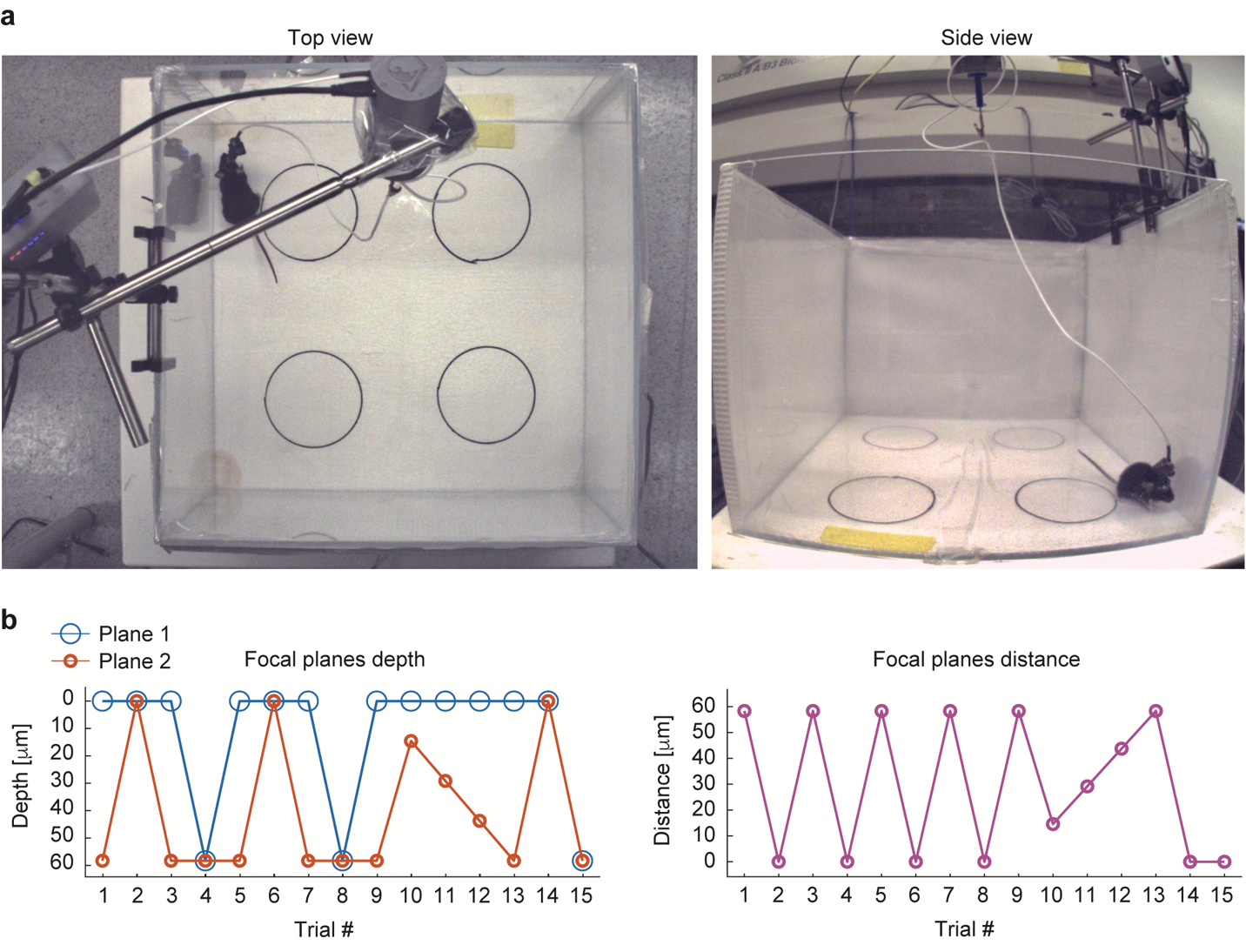
**

**Supplementary Figure 1 | In vivo open field recordings.** (**a**) Sample images from top camera (left) and side camera (right) during an open field trial. (**b**) Positioning of the two focal planes during each open field trial, showing the estimated focal plane depth (left) and the corresponding distance between the two alternating focal planes (right); for each mouse, depth 0 is defined as the depth of the most shallow focal plane.

**
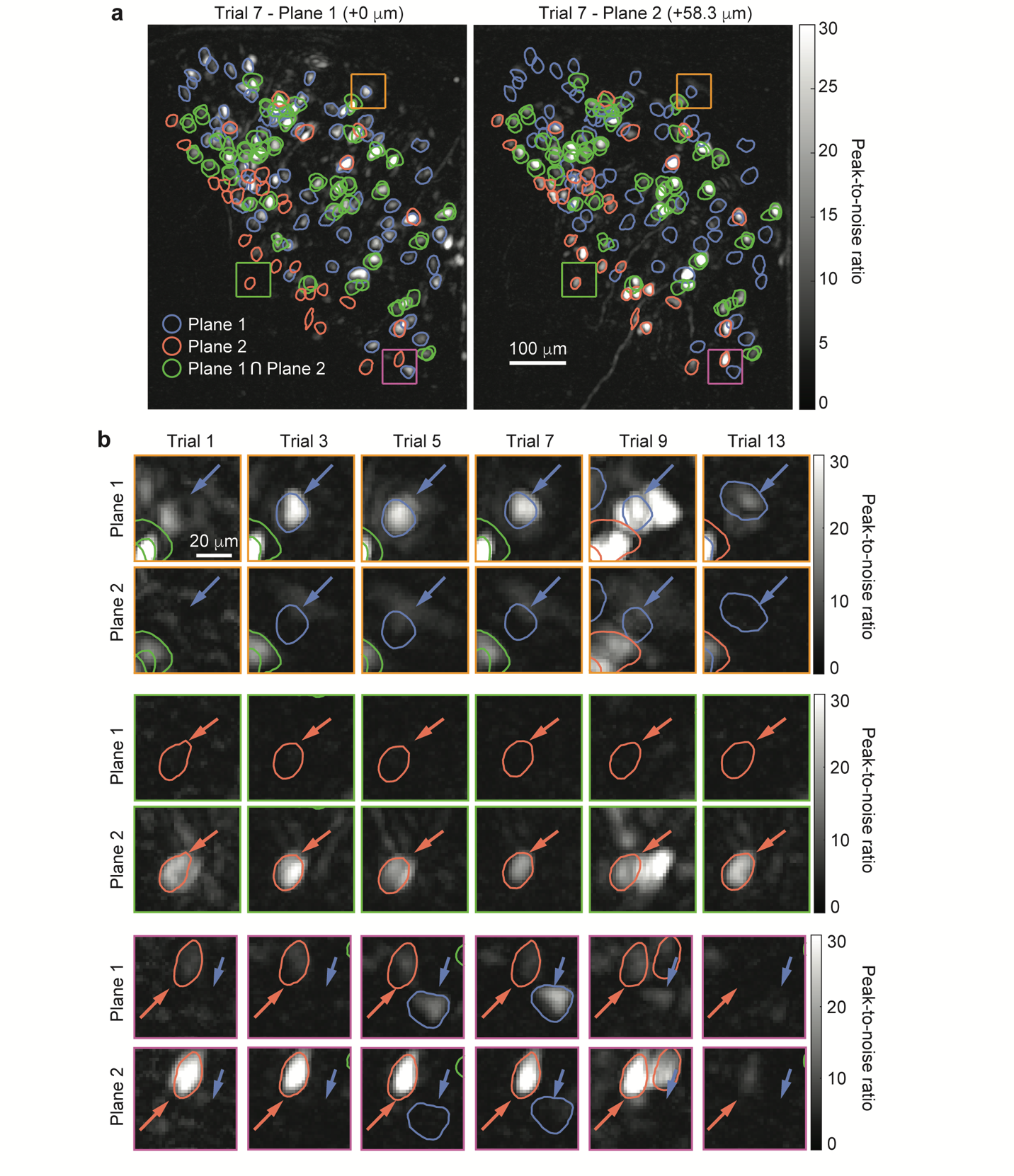
**

**Supplementary Figure 2 | Representative cell map.** (**a**) Cell maps for a representative trial, highlighting neurons detected only on Plane 1 (blue), only on Plane 2 (red) and on both planes (green). (**b**) Zoomed-in detail of the PNR maps for the 3 representative regions demarcated by the colored squares in **a**, across all trials with largest focal plane difference. Top and bottom rows for each of the 3 groups represent Plane 1 and Plane 2, respectively. Colored arrows point at the position of corresponding color-coded neurons.

**Supplementary Movie 1 | Focal plane change for representative trial in Fig. 4**. Calcium imaging video for the first 50 s of the representative trial depicted in Fig. 4, showing background subtracted videos with overlayed cell maps for the unparsed video (both planes alternating, left), plane 1 only (middle), and plane 2 only (right).
